# From RAAS blockade to regenerative medicine: evolving treatment strategies in Alport syndrome

**DOI:** 10.1007/s00467-025-07046-z

**Published:** 2025-11-16

**Authors:** Claudia Lo Re, Jin-Ju Kim, Alessia Fornoni

**Affiliations:** 1 Katz Family Division of Nephrology and Hypertension, Department of Medicine, Miller School of Medicine, University of Miami, 1580 NW 10th Ave, Miami, FL 33136 USA; 2 Peggy and Harold Katz Family Drug Discovery Center, University of Miami, Miller School of Medicine, Miami, FL USA; 3 Unit of Nephrology and Dialysis, Department of Clinical and Experimental Medicine, A.O.U. “G. Martino”, University of Messina, Via Consolare Valeria N1, Bldg B, Floor 3, Messina, 98125 Italy

**Keywords:** Alport syndrome, Proteinuria, Gene therapy, RAAS inhibition, Sparsentan, Mitophagy, New treatment

## Abstract

Alport syndrome (AS) is a hereditary glomerulopathy caused by mutations in the *COL4A3*, *COL4A4*, or *COL4A5* genes, leading to progressive kidney decline and extrarenal manifestations. Advances in genetic testing have enabled the reclassification of AS into X-linked, autosomal recessive, and autosomal dominant forms, facilitating more accurate diagnosis and risk stratification. While renin-angiotensin-aldosterone system (RAAS) blockade remains the foundation of treatment to delay kidney failure, it does not directly target the underlying molecular pathology. Adjunctive commercially available metabolic modulators, including SGLT2i, mineralocorticoid receptor antagonists, ezetimibe and GLP-1 receptor agonists, may offer additional kidney protection. Ameliorating therapies being tested in Phase II trials include endothelin receptor antagonists (e.g., atrasentan), dual endothelin receptor antagonist and angiotensin II receptor inhibition (e.g., sparsentan) FXR agonists (e.g., vonafexor), inducers of cholesterol efflux (e.g., VAR200 and R3R01), and NOX1/4 inhibitors (e.g., setanaxib), several of which are currently being evaluated in clinical trials. Novel strategies such as exon skipping, gene editing, and nonsense mutation readthrough (e.g., ELX-02) are advancing toward precision medicine approaches as disease modifying agents targeting the genetic cause of AS. Moreover, therapies targeting mitochondrial function, such as mitophagy enhancers, have demonstrated preclinical promise. Stem cell-based approaches are also being explored for their regenerative and anti-fibrotic effects. This review summarizes the current landscape of AS classification and treatment, highlighting both standard interventions and experimental therapies. Emphasis is placed on the molecular mechanisms underlying podocyte injury and fibrosis, recent preclinical findings, and ongoing clinical trials that may shift future therapeutic paradigms.

**Graphical abstract:**

**Figure:**
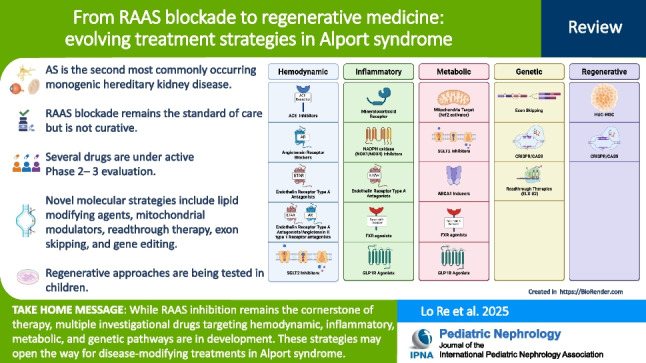
A higher resolution version of the Graphical abstract is available as Supplementary information

**Supplementary information:**

The online version contains supplementary material available at 10.1007/s00467-025-07046-z.

## Introduction

Alport syndrome (AS) is the second most commonly occurring hereditary kidney disease after autosomal dominant polycystic kidney disease (ADPKD). It is a progressive hereditary nephropathy caused by mutations in the *COL4A3*, *COL4A4*, and *COL4A5* genes, which encode the α3, α4, and α5 chains of type IV collagen [1], a crucial structural component of the glomerular basement membrane (GBM) [2], cochlea [3], and lens capsule [4]. Disruption of GBM architecture results in persistent hematuria from early childhood, progressive proteinuria, and, in many cases, an inevitable progression to kidney failure. Extrarenal manifestations include bilateral sensorineural hearing loss [3] and ocular abnormalities [4] such as anterior lenticonus and perimacular fleck retinopathy [5]. The current classification of AS is primarily based on the mode of inheritance and molecular profile. X-linked Alport syndrome (XLAS) [6], due to mutations in *COL4A5*, occurs in approximately 1:2000 individuals (2:1 F/M) [7] and exhibits a more severe phenotype in males, with CKD stage 5 typically occurring between the second and third decades of life [6]. Autosomal recessive Alport syndrome (ARAS), caused by biallelic mutations in *COL4A3* or *COL4A4*, results in a clinical presentation comparable to XLAS in males [8]. Autosomal dominant Alport syndrome (ADAS), more recently delineated through next-generation sequencing (NGS), is much more frequent (1:100) [9] and presents with a generally milder but variably penetrant course [10, 11]. However, the real prevalence of AS is underestimated based on recent genotype-first and population-based studies, given the high frequency of predicted pathogenic *COL4A3–COL4A5* variants in large sequencing cohorts. Moreover, heterozygous *COL4A3*/*4* or *COL4A5* variants can sometimes present with proteinuria and histological features mimicking focal segmental glomerulosclerosis (FSGS), leading to diagnostic challenges and highlighting the need for genetic testing in such cases [7, 9, 12]. In pediatric nephrology, early detection of AS is crucial, as timely therapeutic intervention can significantly delay disease progression. Despite advances in molecular diagnostics and genotype-phenotype correlations, the therapeutic landscape remains limited, with renin-angiotensin system (RAS) blockade being the cornerstone of treatment [13]. However, disease progression typically continues, emphasizing the unmet need for targeted and disease-modifying therapies.

This review explores current standard-of-care practices, evaluates emerging therapeutic strategies, and discusses ongoing clinical trials and novel targets.

## Current standard of care

To date, there is no drug formally approved by regulatory agencies specifically for the treatment of AS. Nevertheless, according to the recently published ERKNet/ERA/ESPN 2024 clinical practice guideline on AS, the recommended standard of care consists of early initiation of renin-angiotensin-aldosterone system (RAAS) inhibition to reduce albuminuria and delay kidney function decline [14].

## Renin-angiotensin-aldosterone system (RAAS) inhibition

Ramipril is the first drug officially used for AS. It belongs to the class of drugs called angiotensin-converting enzyme (ACE) inhibitors, which improve intraglomerular pressure and glomerular hyperfiltration by blocking the RAAS and consequently reducing the loss of protein in the urine. Gross et al. in 2012 conducted studies on the safety and efficacy of ramipril in children with favorable results. The EARLY PROTECT trial showed that use of ramipril with AS aged > 2 years when there is either isolated microscopic hematuria or microalbuminuria (defined as 30–300 mg albumin/g creatinine) is safe and effective compared to placebo [13]. The use of ramipril is recommended in the early stages of the disease because it improves life expectancy, and it slows kidney disease progression by 50% [13, 15, 16]. Particularly, ramipril can be safely started at the time of diagnosis in ≥ 24 months males with XLAS and in all patients with ARAS. In females with XLAS and in males and females with ADAS, therapy can be started at the onset of microalbuminuria [17].

## Emerging and experimental therapies

**Fig. 1:**
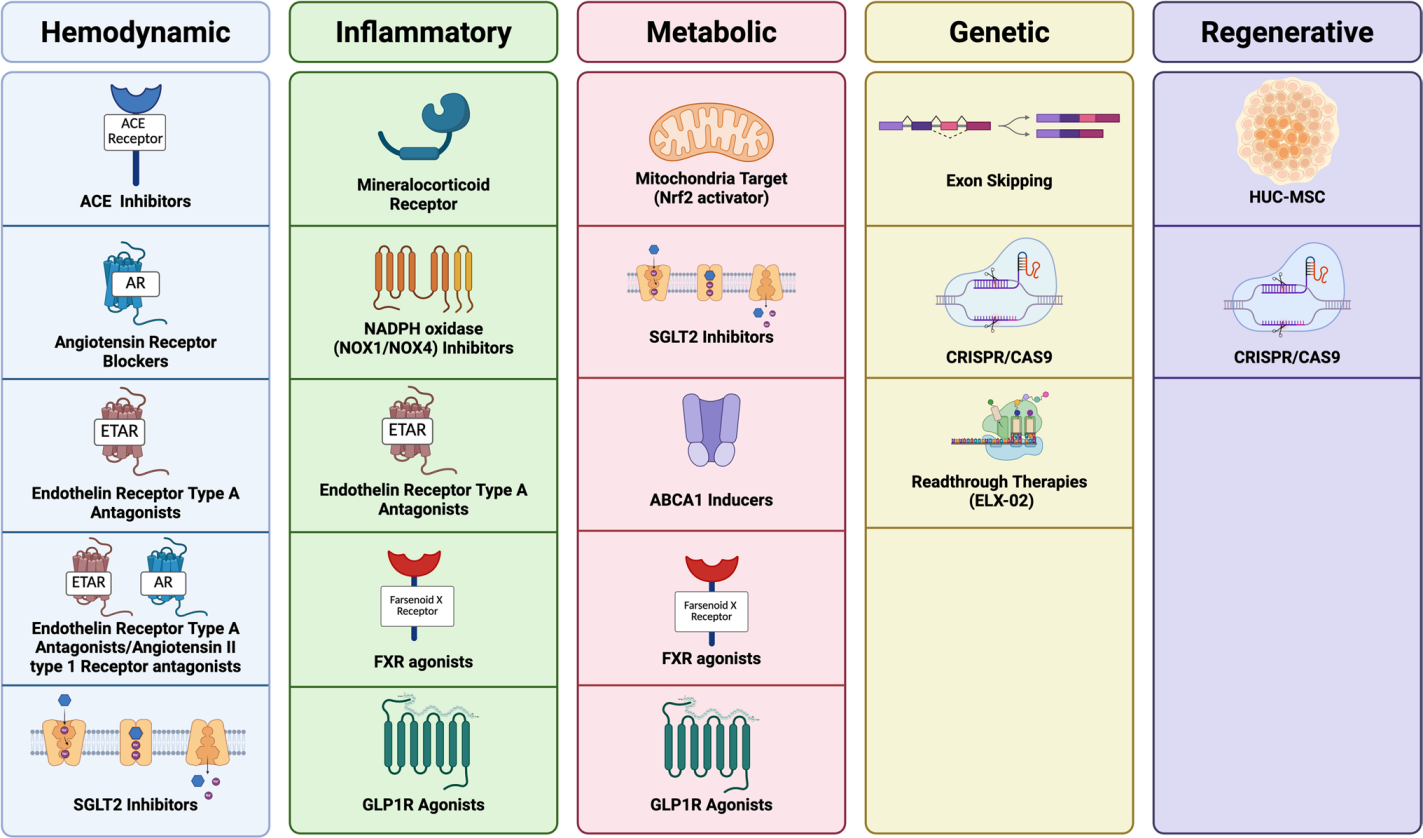
Therapeutic areas and respective targets in Alport syndrome. The figure summarizes current and emerging therapeutic approaches to Alport syndrome, organized by therapeutic area. Genetic/molecular strategies include exon skipping, readthrough therapy (ELX-02), and gene editing approaches (e.g., CRISPR/Cas9). Hemodynamic therapies aim to reduce intraglomerular pressure and proteinuria using ACE inhibitors/ARBs and endothelin A receptor antagonists (e.g., sparsentan, atrasentan). Inflammation is targeted by mineralocorticoid receptor antagonists (e.g., finerenone), endothelin receptor antagonists, NOX1/NOX4 inhibitors (e.g., setanaxib), FXR agonists (e.g., obeticholic acid, vonafexor), and GLP-1 receptor agonists. Metabolic modulators address podocyte lipid handling and mitochondrial health, including ABCA1 inducers (R3R01), SGLT2 inhibitors (e.g., dapagliflozin, empagliflozin), and mitochondrial modulators (e.g., TJ0113). Some compounds, such as endothelin receptor antagonists, SGLT2 inhibitors, and FXR agonists, are represented in multiple categories due to their pleiotropic effects. Finally, regenerative strategies include human umbilical cord mesenchymal stem cells (hUC-MSCs), which may contribute to tissue repair through differentiation, paracrine signaling, and immunomodulation. CRISPR/Cas9 may also be considered a potential future regenerative approach. Created in https://BioRender.com

The limitations of standard RAAS blockade in halting the progression of AS [13] have driven research into novel, targeted therapies aimed at modifying disease mechanisms or ameliorating disease progression. Below we highlight several Phase 2 and 3 interventional trials, currently ongoing and recently completed in AS, targeting different pathogenic pathways. These studies are summarized in Table 1 and illustrated in Fig. 1, which provide an overview of the investigational drugs, their mechanisms of action, and trial status.

**Table 1:** Summary of Ongoing and recently Completed Clinical Trials for AS This table provides an overview of interventional studies currently evaluating or recently completing evaluation of investigational therapies in AS, including mechanism of action, target population, and study status

Study name	Drug	Mechanism	Population	Status	Sponsor
EPPIK	Sparsentan	Dual ETA/AT1 receptor blocker	Pediatrics & Adolescents	Recruiting (Phase 2)	Travere Therapeutics
ALPESTRIA-1	Vonafexor	FXR agonist	Adolescents & Adults	Active, not recruiting (Phase 2)	ENYO Pharma
ELX-02	ELX-02	Read-through compound (COL4 mutations)	Pediatrics & Adults	Ongoing (Phase 2)	Eloxx Pharmaceuticals
FIONA	Finerenone	Non-steroidal MRA	Pediatrics & Adults	Recruiting (Phase 3)	Bayer
R3R01-201	R3R01	Mediates cholesterol efflux by ABCA1 induction	Adolescents & Adults	Completed (Phase 2)	River 3 Renal Corporation
VAR200-0301	2HPβCD	Mediates cholesterol efflux	Adults	Ongoing (Phase 2)	ZyVersa
hUC-MSC	hUC-MSC	Stem cell therapy (anti-inflammation)	Pediatrics & Adults	Planned (Phase 2)	Guangzhou Women& Children’s Medical Center
Setanaxib	Setanaxib	NOX1/4 inhibitor (anti-fibrotic)	Adolescents & Adults	Completed (Phase 2)	Calliditas Therapeutics
AFFINITY	Atrasentn	Selective ETA receptor antagonist	Adolescents & Adults	Active, not recruiting (Phase 2)	Novartis Pharmaceuticals
CARDINAL	Bardoxolone methyl	Nrf2 activator	Adolescents & Adults	Completed, negative (Phase 3)	Biogen
EAGLE	Bardoxolone methyl	Nrf2 activator (long-term safety)	Adults	Terminated (Phase 3)	Biogen
HERA	Lademirsen (anti-miR-21)	microRNA inhibitor	Adolescents & Adults	Terminated for futility	Sanofi
DOUBLE PRO-TECT Alport	Dapagliflozin	SGLT2 inhibitor	Adolescents & Young Adults	Recruiting (Phase 3)	German Research Foundation DFG

## RAAS-adjacent and metabolic modulators

### Sodium-glucose cotransporter-2 (SGLT2) inhibitors

Sodium-glucose cotransporter-2 (SGLT2) inhibitors act by blocking the sodium-glucose cotransporter-2 in the proximal tubule of the kidney, reducing glucose and sodium reabsorption. This leads to increased glucose excretion in the urine, improved glycemic control, and lower intraglomerular pressure [18].

Originally developed for glycemic control in type 2 diabetes mellitus (T2DM), SGLT2 inhibitors have demonstrated kidney protective effects that are independent of glucose lowering, mediated by anti-inflammatory, anti-fibrotic, and hemodynamic mechanisms. These properties have led to growing interest in their use in AS, but pre-clinical evidence remains mixed.

A recent preclinical study demonstrated that empagliflozin alleviates podocyte lipotoxicity and improves kidney function in experimental AS, primarily by shifting cellular metabolism from glucose toward fatty acid oxidation [19]. In AS mouse models, empagliflozin reduced albuminuria, preserved podocyte number, and lowered kidney lipid accumulation, highlighting podocyte lipid modulation as a potential therapeutic target in AS [19]. In the clinical setting, a significant contribution to our understanding of SGLT2 inhibitor use in AS comes from an international, multicenter, real-world observational study (NCT02378805), which enrolled 112 patients with genetically confirmed AS. The primary endpoint was the change in albuminuria after six months of therapy. Most patients (96%) were treated with dapagliflozin, while a smaller subset (4%) received empagliflozin**,** reflecting real-world prescribing patterns and availability at the time of the study. Although reductions in albuminuria were observed, the study also reported a modest decline in estimated glomerular filtration rate (eGFR), raising questions regarding long-term kidney safety of off-label SGLT2 inhibitor use in this population [20]. These findings underscore the need for controlled clinical trials, particularly in pediatric and genetically defined populations, to determine the efficacy and safety of individual SGLT2 inhibitors in AS management. The DOUBLE PRO-TECT Alport trial (NCT05944016) is the first placebo-controlled RCT specifically designed to test a sodium-glucose cotransporter-2 inhibitor (SGLT2i) (dapagliflozin) in adolescents and young adults with AS. The primary endpoint is change in urinary albumin-to-creatinine ratio (UACR), with eGFR slope and safety outcomes as key secondary endpoints. This study will provide the first high-quality evidence for the role of SGLT2i in AS management [21, 22].

## Mineralocorticoid receptor blockers (MRA)

Among emerging adjunctive therapies for AS, finerenone, a selective non-steroidal mineralocorticoid receptor antagonist (MRA), has garnered significant interest due to its anti-fibrotic, anti-inflammatory, and antioxidant properties. Finerenone blocks aldosterone-mediated signaling pathways which contribute to kidney fibrosis, vascular dysfunction, and oxidative stress [23]. These mechanisms are central to the progression of AS pathology. Unlike traditional steroidal MRAs, finerenone demonstrates improved tissue selectivity and a lower risk of hyperkalemia [23]. It is already approved for use in adult patients with chronic kidney disease (CKD) and T2DM, based on the results of two large Phase 3 trials (FIDELIO-DKD and FIGARO-DKD), and a pooled analysis (FIDELITY), which showed a 14% reduction in cardiovascular risk and 23% reduction in kidney outcome risk compared to placebo [24]. In the context of AS, preclinical data support finerenone’s potential to reduce kidney inflammation and fibrosis. A study by Zhu et al. using Col4a3−/− mice demonstrated that co-treatment with finerenone, ramipril, and empagliflozin led to improved kidney protection and survival in an AS mouse model [25]. Based on these findings, the FIONA study (NCT05196035) was initiated. This study is a multicenter, randomized, double-blind, placebo-controlled Phase 3 trial designed to evaluate the efficacy, safety, and pharmacokinetics/pharmacodynamics (PK/PD) of an age- and weight-adjusted oral regimen of finerenone in pediatric patients (aged 6 months to < 18 years) with CKD and proteinuria. Participants receive finerenone in combination with ACE inhibitors or angiotensin receptor blockers (ARB) over a 6-month period [26]. The primary endpoint focuses on achieving a ≥ 30% reduction or percent change in urinary protein-to-creatinine ratio (UPCR) by day 180, a validated surrogate marker of kidney damage in children [26]. To assess long-term safety and sustained benefit, the FIONA OLE study (NCT05457283) follows patients from the original trial for an additional 18 months in an open-label extension design. All participants receive finerenone, allowing for evaluation of treatment-emergent adverse events (TEAEs), serum potassium changes, and blood pressure effects over time. Notably, the FIONA program includes feedback from pediatric nephrology networks, caregivers, and ethical advisory boards. This reflects a patient-centered approach tailored to a vulnerable pediatric population [27]. While trial results remain pending, these studies represent a critical effort to evaluate finerenone as a potential disease-modifying therapy for pediatric patients with AS and persistent proteinuria unresponsive to conventional RAAS blockade.

## Glucagon-like peptide-1 antagonist (GLP-1’s)

Glucagon-like peptide-1 receptor agonists (GLP-1 RAs) have demonstrated kidney-protective effects in both diabetic and non-diabetic chronic kidney disease through mechanisms that extend beyond glycemic control. These include anti-inflammatory, anti-fibrotic, and antioxidant effects, which are particularly relevant in progressive fibrotic kidney disease such as AS [28, 29]. A recent review by Boeckhaus et al. (2025) explored the rationale for repurposing GLP-1 RAs as a potential therapeutic strategy for AS, based on their ability to reduce kidney oxidative stress and fibrosis, two key drivers of disease progression in AS. Although no clinical trials have yet specifically evaluated GLP-1 RAs in patients with AS, their biological effects on the kidney suggest a promising future direction for therapeutic development. Further preclinical and clinical studies are warranted to assess safety, efficacy, and disease-modifying potentia**l** of GLP-1RAs, particularly in pediatric and early-stage patients with AS, where interventions aimed at slowing fibrosis may offer long-term benefits [30].

## Endothelin pathway blockade

### Dual endothelin type A receptor (ETAR) and angiotensin II type 1 receptor (AT1R) inhibitors

Sparsentan is a first-in-class dual endothelin type A receptor (ETAR) and angiotensin II type 1 receptor (AT1R) antagonist, recently approved in the United States for the treatment of adult patients with IgA nephropathy (IgAN) at risk of rapid progression. It is currently under evaluation for other rare kidney diseases, including FSGS. Both ETAR and AT1R contribute to vasoconstriction, inflammation, and kidney fibrosis, and their simultaneous inhibition by sparsentan may provide superior renoprotection compared to single pathway blockade [31–33]. The EPPIK study (NCT05003986) is a multicenter, open-label, 112-week Phase 2 trial designed to evaluate the safety and efficacy of sparsentan in children and adolescents with glomerular diseases, including AS [34]. Results from this pediatric study will be important in determining whether dual endothelin-angiotensin blockade may provide added benefit when compared to standard RAAS inhibition alone in the treatment of hereditary glomerulopathies.

## Selective ETAR antagonist

Atrasentan is a selective endothelin type A receptor (ETAR) antagonist that reduces intraglomerular pressure and proteinuria by blocking the vasoconstrictive and pro-fibrotic effects of endothelin, a key mediator of progressive glomerular injury [35]. It has demonstrated kidney-protective effects in proteinuric glomerular diseases and was recently approved by the FDA for the treatment of adults with IgAN at risk of rapid disease progression. The drug is currently being evaluated in the AFFINITY study (NCT04573920), a Phase 2, open-label, basket-design clinical trial assessing its efficacy and safety in various proteinuric glomerular disease [36]. While atrasentan holds promise as a therapeutic agent for glomerular disorders, no data currently support its efficacy in AS, and further studies are needed to determine its potential role in this context.

## Targeting lipid metabolism and fibrosis in AS

### R3R01 and OSBPL7 inhibitors: ABCA1 modulation and cholesterol efflux

ABCA1 (ATP-binding cassette transporter A1) plays a key role in cellular lipid homeostasis by facilitating the efflux of intracellular cholesterol to apolipoprotein A-1 and HDL particles [37]. In AS and other glomerular diseases, ABCA1 expression is downregulated, leading to lipid accumulation, oxidative stress, macrophage infiltration, and interstitial fibrosis [38, 39]. As a result, research is increasingly focusing on drugs targeting cholesterol efflux via ABCA1 as a potential therapeutic strategy [40]. R3R01 is an investigational small molecule designed to enhance ABCA1 activity and restore cholesterol efflux in kidney cells. Preclinical studies suggest that R3R01 reduces kidney parenchymal lipid accumulation, suppresses proinflammatory cytokine expression, and attenuates fibrotic remodeling. R3R01 belongs to a novel class of 5-arylnicotinamides compounds that we identified through a phenotypic drug discovery (PDD) approach as potent inducers of ABCA1 [40].These compounds target Oxysterol Binding protein Like 7 (OSBPL7) and increase ABCA1-dependent cholesterol efflux without transcriptional activation. Unlike liver X receptor (LXR) agonists, which induce ABCA1 mRNA but are limited by side effects such as hypertriglyceridemia, 5-arylnicotinamide elevates ABCA1 protein levels without affecting mRNA expression. In the same class of agents, compound G (Cpd G) demonstrated significant therapeutic effects in both adriamycin-induced nephropathy and Col4A3KO mouse model. Cpd G reduced proteinuria, cholesterol ester accumulation, and glomerular injury, while also extending survival in AS mice [40]. Notably, it outperformed ramipril in mitigating disease severity. These findings position OSBPL7 as a novel therapeutic target in AS and other proteinuric kidney diseases [40].

A Phase 2 clinical trial (NCT05267262) is currently underway to assess the safety and efficacy of R3R01 in patients with AS and FSGS with persistent proteinuria despite ACE inhibitor therapy. Primary endpoints include reductions in proteinuria, improvements in lipid profiles, and changes in biomarkers of fibrosis and inflammation [12, 41]. Although clinical trials have only begun for R3R01, the strong preclinical data for OSBPL7-targeting compounds such as Cpd G support their future translation into clinical use.

## 2- Hydroxypropyl-β-cyclodextrin: a cholesterol efflux mediator

HPβCD is a modified cyclodextrin which selectively binds and sequesters lipophilic molecules such as unesterified cholesterol, phospholipids, and hydrophobic protein fragments. HPβCD facilitates the efflux of intracellular cholesterol by extracting it from cellular membranes and forming water-soluble inclusion complexes [42, 43]. We found that HPβCD protects AS mice (Col4A3KO) from developing proteinuria, progressive kidney failure, and fibrosis [44]. In addition, HPβCD treatment resulted in improved survival. Similarly, HPβCD protected mice with adriamycin-induced nephropathy from proteinuria, elevated blood urea nitrogen (BUN), and mesangial expansion. Moreover, HPβCD’s protective effect in AS mice was associated with reduced lipid accumulation and it was found that HPβCD reduced kidney cholesterol ester, lipid droplet, and cholesterol crystal content. These findings support our previous study [45] which demonstrated a preventive effect of HPβCD in diabetic kidney disease (DKD) progression and in a newly established model of experimental ApoL1-driven FSGS. Based on these findings, a Phase 2 open label study is ongoing to evaluate the clinical efficacy and safety of 1 dose level of 2-hydroxypropyl-β-cyclodextrin (2-HPβCD) given intravenously in adult patients with T2DM with DKD and proteinuria [46]. Although HPβCD has shown protective effects in preclinical models of AS, including Col4A3 KO mice, no clinical studies have yet been conducted in patients with AS. Currently, the therapeutic potential of HPβCD is being evaluated only in a clinical trial involving patients with DKD, highlighting the need for future clinical translation in other proteinuric kidney diseases such AS.

## FXR agonist

Vonafexor is a highly selective non-steroidal agonist of farnesoid X receptor (FXR), a nuclear receptor that regulates lipid metabolism, inflammation, and fibrosis [47]. In AS mouse models, kidney FXR activity is significantly reduced. Pre-clinical studies demonstrated that activating FXR with obeticholic acid (OCA) improved nephroprotective effects in AS model, leading to reductions in BUN, plasma creatinine, UACR, and kidney fibrosis. This beneficial effect is mechanistically linked to the modulation of neutrophil extracellular traps (NETs) and sphingosine-1-phosphate (S1P) signaling [48]. Notably, extensive NETosis has been observed in both AS mouse kidneys and human AS kidney biopsies, with severity of NETosis correlating positively with clinical markers of kidney disease such as serum creatinine, interstitial fibrosis and tubular atrophy (IFTA), and glomerulosclerosis . S1P, a pro-inflammatory lipid, stimulates NETosis, and in AS mice, kidney S1P levels and the expression of its producing enzyme, Sphk1, are elevated [48]. Crucially, FXR agonism represses Sphk1 expression and reduces S1P production, thereby mitigating kidney neutrophilic inflammation and NETosis, as further confirmed by the therapeutic effect of myriocin (an S1P synthesis inhibitor) in AS mice. These findings suggest that targeting the FXR-S1P-NETosis pathway could be a promising new therapeutic strategy for AS by preventing kidney inflammation and disease progression. It is important to note that the previously discussed vonafexor, while an FXR agonist, is investigated for NASH and is not directly covered in the provided source as a treatment for AS [47].

While vonafexor is a selective non-steroidal FXR agonist, no pre-clinical data in AS have been published so far. Nonetheless, based on the rationale provided by OCA studies, vonafexor was granted Orphan Drug Designation (ODD) by the FDA and EMA for AS in 2023. A Phase 2 trial, ALPESTRIA-1 (NCT06425055), launched in 2024 is currently evaluating the safety and efficacy of once-daily oral vonafexor in 20 patients with genetically confirmed AS at high risk of progression. The study includes a 24-week treatment period followed by a 12-week follow-up. The study aims to determine vonafexor’s potential to modify disease trajectory through FXR-mediated antifibrotic and anti-inflammatory effects [49]. However, as we recently demonstrated that targeting S1P content in kidney cortexes may not translate into improvement of eGFR, the long-term beneficial effects of this drug remain to be established [50].

## Ezetimibe: modulating fatty acid uptake and mitochondrial lipid handling

Ezetimibe, a cholesterol absorption inhibitor that blocks Niemann-Pick C1 like1 (NPC1L1) protein in the small intestine [51], has shown compelling effects beyond lipid lowering, including kidney protection in proteinuric kidney diseases. In AS, podocytes demonstrate increased fatty acid (FA) uptake and reduced LD (lipid droplets)-mitochondria contact formation, resulting in lipotoxic stress, oxidative damage, and impaired mitochondrial ATP production. We demonstrated that ezetimibe reduces FA uptake by inhibiting the DDR1–CD36 interaction, thereby limiting intracellular lipid overload [52]. Furthermore, it enhances the formation of LD-mitochondria contacts, facilitating efficient FA trafficking and oxidation in mitochondria. These actions improve mitochondrial membrane potential, increase ATP production, and mitigate oxidative stress, ultimately preserving podocyte structure and function [53]. Although current data are limited to preclinical models, they lay the groundwork for future clinical translation. In DKD, a recent study by Heinrich et al. [54] demonstrated that ezetimibe decreased kidney lipid content and improved metabolic biomarkers, suggesting its translational potential in other lipid driven glomerulopathies, including AS. Ezetimibe has also been shown to reduce markers of inflammation and fibrosis in obese and hyperlipidemic animal models, likely through modulation of lipid-sensing pathways such as SREBP and AMPK signaling. While current clinical evidence is limited, the dual action of ezetimibe on lipid absorption and intracellular FA metabolism provides a compelling rationale for further investigation in clinical trials targeting AS and related hereditary nephropathies.

## NOX1/4 inhibitor: targeting oxidative stress and fibrosis

Targeting oxidative stress and fibrosis, preclinical studies exploring the therapeutic potential of NADPH oxidase (NOX) inhibitors in kidney disease such as AS and diabetic nephropathy point toward a common underlying cellular mechanism involving the reduction of oxidative stress and its downstream effects on inflammation and fibrosis. Setanaxib is the first selective inhibitor of NADPH oxidase isoforms NOX1 and NOX4, key enzymes that contribute to reactive oxygen species (ROS) generation and kidney fibrosis [55]. A preclinical study, displayed as an abstract at the American Society of Nephrology (ASN) meetings in 2024, in the Col4a3 KO mouse model of AS investigated setanaxib which, particularly when combined with the ACE inhibitor ramipril, led to a further reduction in urinary albumin levels and albumin/creatinine ratio compared to ramipril alone [56]. Histologic analysis showed that this combination treatment significantly decreased glomerular sclerosis and overall fibrosis. Furthermore, proteomic and in silico analyses indicated increased detection of glomerular basement membrane and collagen proteins with the combined treatment [56]. These findings underscore that the setanaxib/ACEi combination induces mechanisms that reduce the decline in glomerular function and fibrosis but remain preliminary and not yet published in peer-reviewed form. More detailed insights into NOX inhibition mechanisms come from diabetic nephropathy models, where dual NOX1/4 inhibitor (setanaxib, GKT137831), reduced NADPH-dependent ROS production, extracellular matrix protein accumulation, podocyte loss, and macrophage infiltration [55]. These findings suggest that setanaxib’s renoprotective effects in AS may similarly stem from mitigating oxidative stress and its fibrogenic and inflammatory consequences, but direct evidence is still limited, and further research is needed. While setanaxib has advanced to clinical trials for T2DM and fibrotic diseases, trials in AS have not yet begun. However, the compelling preclinical efficacy in AS mouse models provides a strong rationale for expanding NOX-targeted therapies into hereditary glomerular disorders with oxidative stress components. Based on these findings, Calliditas Therapeutics initiated a Phase 2 randomized, double-blind, placebo-controlled trial (NCT06274489) in November 2023. This study evaluates the safety and efficacy of setanaxib in approximately 20 AS patients with persistent proteinuria despite optimized RAAS blockade. Primary endpoints include changes in urine protein-to-creatinine ratio (UPCR) and eGFR, assessing both functional and structural kidney outcomes. The study has been completed in 2025, but results are not yet available [57].

## Mitochondrial modulation therapy

Mitochondria play an essential role in kidney energy metabolism and redox balance, particularly in proximal tubular and glomerular epithelial cells. In AS, mitochondrial dysfunction, ROS accumulation, and defective mitophagy contribute to progressive kidney injury [53, 58]. Mitophagy, the selective autophagic process targeting damaged mitochondria, is critical for maintaining mitochondrial quality and preventing apoptosis [58]. Preclinical studies have demonstrated that impaired mitophagy leads to the accumulation of dysfunctional mitochondria and excessive production of ROS, which further exacerbates cellular injury and promotes tubulointerstitial fibrosis [58]. Notably, Lu et al. demonstrated that pharmacologic activation of mitophagy via AMPK or the PINK1/Parkin pathway restored mitochondrial structure, decreased ROS generation, and improved kidney histology in mice with experimental AS [59]. Based on the understanding of these mechanisms, agents targeting mitochondrial quality and mitophagy were under clinical evaluation for AS [60]. One such agent, bardoxolone methyl (BARD), which activates Nrf2, was evaluated for AS in the Phase 3 CARDINAL trial (NCT03019185) [61–63]. Nrf2 has been shown to upregulate mitophagy by targeting PINK1, thereby enhancing mitochondrial quality control [64]. However, the Food and Drug Administration (FDA) determined there was insufficient evidence of BARD’s safely and efficacy for this indication. Furthermore, a Phase 3 trial involving BARD in patients with ADPKD/CKD was terminated in May 2023 as it did not significantly reduce the risk of kidney failure development, suggesting that indirect mitochondrial mediation via Nrf2 activation might be suboptimal [65, 66]. More recently, TJ0113, a compound specifically described as being able to induce mitophagy, was described in preclinical studies to selectively eliminate damaged mitochondria and restore cellular energy metabolism through activation of the PINK1/Parkin pathway [67, 68]. This highlights the emerging potential of directly targeting mitophagy pathways as a therapeutic strategy for AS, building on the preclinical understanding that mechanisms like PINK/Parkin-medicated mitophagy are critical for maintaining mitochondrial health and protecting kidney cells [58]. While early research on mitophagy-centered therapies holds promise, most approaches have yet to reach broad clinical application. The termination of the BARD programs underscores the need for effective, direct mitochondrial targeting strategies. TJ0113 represents a candidate being actively investigated for AS, marking a noteworthy new direction in targeting mitochondrial quality control for kidney protection but no studies in AS are currently registered and no FDA approval exists.

## Genetic, protein repair, and cell-based strategies

### ELX-02: readthrough therapy

Nonsense mutations in *COL4A3*, *COL4A4*, and *COL4A5* account for a significant proportion of pathogenic variants in AS, particularly in X-linked and autosomal recessive forms. These mutations introduce premature termination codons (PTCs), leading to truncated and non-functional type IV collagen chains, thereby compromising the integrity of the GBM and accelerating kidney failure, often by late adolescence. Among emerging genotype-specific therapeutic approaches, ELX-02 represents a promising readthrough agent designed to restore the expression of full-length collagen proteins by selectively overriding nonsense mutations, as reported in a non–peer-reviewed ASN abstract [69, 70]. ELX-02 is a synthetic aminoglycoside analog developed to promote ribosomal readthrough of PTCs while sparing native stop codons. It is preferentially taken up by kidney cells expressing megalin, a scavenger receptor abundant in podocytes and proximal tubular epithelial cells, which facilitates targeted delivery to relevant kidney compartments. Upon uptake, ELX-02 interacts with the eukaryotic ribosomal decoding site, allowing near-cognate tRNA incorporation at the PTC site, potentially enabling the production of a functional full-length protein [71]. A Phase 2 open-label pilot study (NCT05448755/EL-014) is currently underway to evaluate the safety, tolerability, and preliminary efficacy of ELX-02 in AS patients harboring nonsense mutations. This multicenter study is enrolling up to eight participants aged 6 to 30 years with confirmed X-linked or autosomal recessive AS. Eligible participants must carry a documented nonsense mutation in *COL4A5* (for males) or *COL4A3/COL4A4* (for both sexes), specifically UAG or UGA variants, and meet kidney function criteria (eGFR > 60 mL/min/1.73 m^2^, UPCR ≥ 500 mg/g). Participants must also be on a stable ACE inhibitor or ARB regimen for at least four weeks prior to enrollment. Exclusion criteria include history of organ transplantation, autosomal dominant AS, significant liver disease, cardiac dysfunction (LVEF ≤ 40%), or prior dialysis [69]. The treatment protocol involves subcutaneous administration of ELX-02 at 0.75 mg/kg/day for 8 weeks, followed by a 12-week post-treatment observation period. As of this writing, the trial is actively recruiting at three sites: Monash Medical Center (Australia), Great Ormond Street Hospital (UK), and Royal Free Hospital (UK). So far, only preliminary data have been disclosed, mainly through conference abstracts and company press releases, without peer-reviewed full publications [70]. These findings must therefore be interpreted with caution and regarded as exploratory evidence rather than definitive proof of efficacy. Three patients with autosomal recessive AS, all harboring the COL4A4:p. (Ser969Ter) nonsense mutation, were treated with the same ELX-02 regimen. Post-treatment analyses revealed segmental re-expression of the collagen IV α5 chain in glomeruli, indicating successful PTC readthrough and protein production [70]. Additionally, filtration slit density (FSD) improved in all patients, rising from pre-treatment values of 38.8–74.8% (relative to healthy controls) to 72.8–85.0% after 8 weeks. Foot process width (FPW), a marker of podocyte injury, decreased in two of three patients, and proteinuria was reduced in two participants [70]. Importantly, no significant safety signals were observed, supporting the tolerability of ELX-02. At present, these findings must be regarded as exploratory and not definitive evidence of efficacy of a gene-targeted therapy in a podocyte-specific Mendelian disease but underscore the therapeutic potential of readthrough strategies for nonsense mutation-associated kidney disorders. While further randomized, placebo-controlled studies are required to confirm clinical benefit, these early experiments support the broader utility of readthrough therapy in AS and potentially other genetic kidney diseases with nonsense mutations expressed in megalin-positive cell types. The development of ELX-02, supported by Eloxx Pharmaceuticals, may open a new frontier in precision nephrology by targeting the genetic root cause of disease in select patient subsets [69]. To place ELX-02 in the broader context of emerging genotype-specific therapies, it is important to note that exon skipping with antisense oligonucleotides has shown encouraging preclinical results in *COL4A5* knockout models but still faces major translational barriers such as efficient kidney delivery and the need for repeated dosing [71]. Likewise, gene editing approaches (e.g. CRISPR/Cas9) offer the theoretical opportunity for permanent correction but remain at the proof-of-principle stage in organoids and animal models, with unresolved challenges including off-target effects and delivery [72–74]. Taken together, ELX-02 represents the only genotype-specific strategy currently in clinical testing for AS, whereas exon skipping and gene editing remain at earlier translational or preclinical stage.

## Exon skipping and gene editing

Exon skipping is a gene therapy approach that aims to restore the reading frame of mutant transcripts by selectively excluding specific exons during pre-mRNA splicing. In the context of AS, pathogenic variants in *COL4A3, COL4A4*, or *COL4A5* often result in truncating mutations, such as nonsense mutations or frameshifts, which introduce premature stop codons. Using antisense oligonucleotides (ASOs), exon skipping therapy can bypass the mutant exon during RNA processing, producing a shorter but in-frame mRNA transcript. This allows for the translation of internally truncated collagen chains that can still assemble into functional or partially functional collagen heterotrimers. Although these proteins may not be identical to the wild-type version, even partial restoration of collagen IV chain assembly and secretion can improve GBM stability and mitigate disease progression.

The therapeutic potential of exon skipping in AS was first demonstrated in a Col4a5 mutant mouse model carrying a nonsense mutation in exon 21 (c.1411C > T, p. Arg471*) [72]. Systemic subcutaneous administration of ASO-ENA, an antisense oligonucleotide targeting exon 21, led to effective exon skipping in kidney tissue. Treated mice showed restored α5(IV) expression on tubular basement membranes and partial recovery on the GBM, as well as re-expression of α3(IV) and α4(IV), indicating successful reassembly of the α3α4α5(IV) heterotrimer. Histological analysis revealed markedly improved kidney architecture, with reductions in glomerulosclerosis, interstitial fibrosis, and inflammatory cell infiltration. Electron microscopy confirmed attenuation of GBM thickening and lamellation. Functionally, ASO-treated mice exhibited significantly lower UACR and reduced serum levels of BUN and creatinine. Most notably, survival was significantly extended compared to untreated controls [72]. Biodistribution studies showed that ASOs accumulated in podocytes and tubular epithelial cells and remained detectable for up to two weeks, suggesting the feasibility of intermittent dosing regimens [72].

To evaluate the applicability of this approach to human systems, researchers utilized kidney organoids derived from induced pluripotent stem cells (iPSCs) from an AS patient carrying a *COL4A5* exon 24 nonsense mutation [73]. These organoids (K001 line) successfully formed nephron-like structures but lacked detectable expression of the *COL4A5* C-terminus, consistent with the mutation [73]. In vitro treatment with exon 24-targeting ASOs restored C-terminal expression of *COL4A5*, with no adverse effects on nephron development, supporting the efficacy of exon skipping in a human genetic background [73]. However, when these organoids were transplanted into immunodeficient mice to promote glomerular vascularization and then treated with ASOs in vivo, the results were less encouraging. Despite the formation of vascularized glomeruli and the presence of mouse-derived vessels, ASO administration via intraperitoneal injection failed to restore C-terminal *COL4A5* expression [73]. Furthermore, there was no significant improvement in slit diaphragm structure, GBM thickness, or lamina densa formation [73]. Gene therapy using adeno-associated virus (AAV) vectors encoding *COL4A5* or *COL4A3* has been explored in animal models, aiming to restore normal *COL4* production in GBM [74]. Although vector size constraints and immune responses remain technical barriers, Heikkilä et al. demonstrated for the first time that adenovirus-mediated transfer of human *COL4A5* cDNA into the pig kidney in vivo led to successful expression and deposition of *COL4A5* chain in the GBM [74]. These findings underscore that in vivo delivery, metabolism, and organoid immaturity remain key challenges to therapeutic success.

As of now, no clinical trials have tested exon skipping therapy in patients with AS. Nevertheless, the promising preclinical outcomes in mouse models and patient-derived kidney organoids provide a strong rationale for further development. Several critical barriers must be addressed before clinical translation can be realized. One of the major limitations is drug delivery and metabolism. Intraperitoneal delivery, used in preclinical models, may result in substantial hepatic metabolism, reducing the amount of ASO that reaches the kidney. Additionally, the glomerular filtration barrier poses a physical constraint, generally allowing only molecules under 50 kDa and less than 10–50 nm in diameter to access podocytes. Targeted intravenous administration or nanoparticle-based delivery systems may improve localization to the kidney and enhance therapeutic efficacy. Another obstacle is the immaturity of current iPSC-derived organoid models. Although they mimic early nephron development, they do not fully recapitulate adult kidney structure or function. This limits their utility in modeling progressive, chronic disease and evaluating long-term therapeutic effects. Even after transplantation, organoids remain developmentally immature and may exhibit aberrant differentiation, such as cartilage formation, or incomplete slit diaphragm development. Efforts to improve organoid vascularization, extend culture duration, or incorporate supportive cell types are ongoing. Lastly, vector limitations present another challenge. While AAV vectors are attractive for their low immunogenicity and stable expression, their packaging capacity (~ 4.7 kb, functionally ~ 2.7 kb) is insufficient for full-length *COL4A3, COL4A4*, or *COL4A5* [74]. Larger vectors like adenovirus have higher capacities but are more immunogenic, and lentiviruses carry risks of genomic integration. In addition, high-dose AAV administration has been associated with systemic immune responses and off-target organ transduction, further complicating delivery strategies [74]. Despite these hurdles, technological advancements in gene editing, delivery systems, and stem cell modeling continue to improve the feasibility of exon skipping and gene therapy for AS [75]. Innovations such as dual-AAV systems to split large genes into multiple vectors or mRNA delivery via lipid nanoparticles may eventually enable safe and effective treatment [75]. Exon skipping thus represents a promising, mutation-specific therapeutic option that may complement or replace current symptomatic treatments for AS in the future. Overall, exon skipping represents a promising mutation-specific therapeutic option for X-linked AS, with strong proof-of-concept efficacy in animal models and encouraging results in patient-derived kidney organoids [71, 72]. However, translation into clinical application is still hampered by major barriers, including the need for efficient and sustained kidney delivery, challenges in metabolism and biodistribution, the immaturity of current organoid models, and the technical limits of vector systems [72–74]. These considerations underscore both the potential and the limitations of exon skipping at its current stage of development, highlighting the need for advances in delivery platforms and preclinical modeling before clinical translation can be realized.

## Stem cell-based therapies

Stem cell-based therapies are under investigation as potential regenerative treatments for AS [76]. Among the most promising are human umbilical cord-derived mesenchymal stem cells (hUC-MSCs), which are attractive due to their accessibility, low immunogenicity, and multipotency. These cells can migrate to sites of injury and contribute to tissue regeneration via differentiation, immune modulation, and paracrine effects. HUC-MSCs have been shown to differentiate into vascular endothelial cells, mesangial-like cells, and even podocyte-like cells, contributing to glomerular repair and restoration of the GBM. Their ability to promote tissue regeneration also relies heavily on paracrine signaling, with secretion of bioactive molecules such as vascular endothelial growth factor (VEGF), hepatocyte growth factor (HGF), and epidermal growth factor (EGF), which enhance angiogenesis, inhibit apoptosis, and suppress inflammation [77]. Beyond direct differentiation, hUC-MSCs exhibit strong immunomodulatory capacity, regulating both innate and adaptive immune responses. They suppress the proliferation of T and B lymphocytes, promote the polarization of macrophages toward an anti-inflammatory phenotype, and reduce secretion of pro-inflammatory cytokines such as TNF-α, IL-6, and IL-17. Importantly, hUC-MSCs have been shown to inhibit the activation of the NLRP3 inflammasome, a central mediator of inflammation in various chronic diseases [76, 77]. Preclinical models of AS have provided critical evidence supporting the therapeutic potential of stem cells. In one of the earliest studies, Sugimoto et al. demonstrated that bone marrow-derived stem cells injected into *COL4A3* knockout mice homed to the glomeruli, differentiated into podocyte-like cells, and contributed to collagen IV production and GBM repair [78]. These mice exhibited reduced proteinuria and improved kidney histology compared to controls [78]. While this study used bone marrow-derived stem cells, subsequent studies have shown similar results using hUC-MSCs. In *COL4A5*-deficient AS models, which more closely resemble X-linked AS in humans, hUC-MSCs administered via the tail vein at a dose of 5 × 10^6^ cells resulted in reduced albuminuria, prolonged survival, and significant attenuation of glomerular inflammation and fibrosis. These therapeutic benefits were attributed to both direct integration of stem cells into kidney structures and indirect paracrine signaling that modified the immune and fibrotic landscape of the kidney [77]. Despite encouraging preclinical findings, clinical translation of stem cell therapy for AS has only recently begun. A Phase I/II clinical trial (NCT06731192) is currently underway at Guangzhou Women and Children’s Medical Center in China to evaluate the safety and efficacy of hUC-MSCs in pediatric patients with AS [79]. This open-label, single-arm study targets children with persistent albuminuria and early-stage chronic kidney disease (CKD stages I–III). Participants receive intravenous infusions of allogeneic hUC-MSCs over several sessions, with primary endpoints focused on safety and changes in albuminuria levels. In addition to routine monitoring, exploratory endpoints in this trial include the collection of blood and urine samples for transcriptomic and proteomic analysis to uncover mechanisms of action such as immunomodulation, anti-fibrotic remodeling, and tissue repair. Notably, the trial does not require immunosuppressive pre-treatment, reflecting prior safety evidence for hUC-MSCs in other kidney conditions. As of the latest update, this study represents the most advanced clinical effort specifically targeting AS with stem cell therapy [76]. Together, these findings suggest that hUC-MSCs hold considerable promise as a disease-modifying treatment in AS. However, further studies with larger cohorts and long-term follow-up are needed to validate their efficacy, optimize dosing and delivery, and establish standardized manufacturing protocols to ensure reproducibility and safety. Preclinical studies suggest that mesenchymal stem cells (MSCs) can migrate into glomeruli, differentiate into podocyte-like cells and contribute to COL4 production and GBM repair [77]. In *COL4A3* KO mice, Sugimoto et al. demonstrated that bone marrow-derived stem cells improved glomerular structures and kidney function [78]. Despite encouraging preclinical data, no clinical trials of stem cell therapy in AS have been initiated, and several challenges remain, including delivery, immunogenicity, and safety concerns.

## Conclusions

Despite the absence of curative therapies, recent advances in understanding the pathogenesis of AS have led to the development of ameliorating promising targeted treatments and novel approaches such as stem cell and gene therapies. Continued research in this direction is essential, and the active participation of patients and patient foundations in clinical trials will be crucial to accelerate progress. Ongoing and future studies will help define the safety, efficacy, and long-term benefits of these emerging therapies, with the goal of improving outcomes and quality of life for individuals living with AS.

## Supplementary information

Below is the link to the electronic supplementary material.

ESM1 Graphical abstract (PPTX 4.64 MB)
