# Correction: From RAAS blockade to regenerative medicine: evolving treatment strategies in Alport syndrome

**DOI:** 10.1007/s00467-025-07087-4

**Published:** 2025-12-11

**Authors:** Claudia Lo Re, Jin-Ju Kim, Alessia Fornoni

**Affiliations:** 1 Katz Family Division of Nephrology and Hypertension, Department of Medicine, Miller School of Medicine, University of Miami, 1580 NW 10th Ave, Miami, FL 33136 USA; 2 Peggy and Harold Katz Family Drug Discovery Center, University of Miami, Miller School of Medicine, Miami, FL USA; 3 Unit of Nephrology and Dialysis, Department of Clinical and Experimental Medicine, A.O.U. “G. Martino”, University of Messina, Via Consolare Valeria N1, Bldg B, Floor 3, 98125 Messina, Italy

**Correction: Pediatric Nephrology**

10.1007/s00467-025-07046-z

The original Table 1 included incorrect information. Below is the original table for reference, followed by the corrected version:

Original Table 1:

**Table 1:** Summary of ongoing and recently completed clinical trials for AS. This table provides an overview of interventional studies currently evaluating or recently completing evaluation of investigational therapies in AS, including mechanism of action, target population, and study status

Study	Drug	Mechanism	Population	Status	Sponsor
**EPPIK**	Sparsentan	Dual ETA/AT1 receptor blocker	Pediatric (1–18yrs)	Completed (Phase 2)	Travere Therapeutics
**ALPESTRIA-1**	Vonafexor	FXR agonist	Adolescents and adults	Completed (Phase 2)	Genfit
**ELX-02**	ELX-02	Read-through compound (*COL4* mutations)	Pediatric (6–30 yrs)	Ongoing (Phase 2)	Eloxx Pharmaceuticals
**FIONA**	Finerenone	Non-steroidal MRA	Pediatric (2–18 yrs)	Recruiting (Phase 3)	Bayer
**R3R01-201**	R3R01	Mediates cholesterol efflux by ABCA1 induction	Adults	Ongoing (Phase 2)	Chinook Therapeutics
**VAR200-0301**	2HPβCD	Mediates cholesterol efflux	Adults	Ongoing (Phase 2)	ZyVersa
**MSC Trial**	hUC-MSC	Stem cell therapy (anti-inflammation)	Not specified	Planned (Phase 2)	Guangzhou Women& Children’s
**Setanaxib Study**	Setanaxib	NOX1/4 inhibitor (anti-fibrotic)	Adults	Completed (Phase 2)	Calliditas Therapeutics
**AFFINITY Study**	Atrasentan	Selective ETA receptor antagonist	Adults	Exploratory (Phase 2)	Chinook Therapeutics
**CARDINAL**	Bardoxolone methyl	Nrf2 activator	Adolescents and adults	Completed, negative (Phase 3)	Reata Pharmaceuticals
**EAGLE**	Bardoxolone methyl	Nrf2 activator (long-term safety)	Adults	Terminated (Phase 3)	Reata Pharmaceuticals
**HERA**	Lademirsen (anti-miR-21)	microRNA inhibitor	Adolescents and adults	Terminated for futility	Sanofi
**DOUBLE PRO-TECT Alport**	Dapagliflozin	SGLT2 inhibitor	Adolescents and young adults	Recruiting	AstraZeneca

Corrected Table 1:

**Table 1:** Summary of Ongoing and recently Completed Clinical Trials for AS This table provides an overview of interventional studies currently evaluating or recently completing evaluation of investigational therapies in AS, including mechanism of action, target population, and study status

Study name	Drug	Mechanism	Population	Status	Sponsor
**EPPIK**	Sparsentan	Dual ETA/AT1 receptor blocker	Pediatrics & Adolescents	Recruiting (Phase 2)	Travere Therapeutics
**ALPESTRIA-1**	Vonafexor	FXR agonist	Adolescents & Adults	Active, not recruiting (Phase 2)	ENYO Pharma
**ELX-02**	ELX-02	Read-through compound (COL4 mutations)	Pediatrics & Adults	Ongoing (Phase 2)	Eloxx Pharmaceuticals
**FIONA**	Finerenone	Non-steroidal MRA	Pediatrics & Adults	Recruiting (Phase 3)	Bayer
**R3R01-201**	R3R01	Mediates cholesterol efflux by ABCA1 induction	Adolescents & Adults	Completed (Phase 2)	River 3 Renal Corporation
**VAR200-0301**	2HPβCD	Mediates cholesterol efflux	Adults	Ongoing (Phase 2)	ZyVersa
**hUC-MSC**	hUC-MSC	Stem cell therapy (anti-inflammation)	Pediatrics	Planned (Phase 2)	Guangzhou Women& Children’s Medical Center
**Setanaxib**	Setanaxib	NOX1/4 inhibitor (anti-fibrotic)	Adolescents & Adults	Completed (Phase 2)	Calliditas Therapeutics
**AFFINITY**	Atrasentn	Selective ETA receptor antagonist	Adults	Active, not recruiting (Phase 2)	Novartis Pharmaceuticals
**CARDINAL**	Bardoxolone methyl	Nrf2 activator	Adolescents & Adults	Completed, negative (Phase 3)	Biogen
**EAGLE**	Bardoxolone methyl	Nrf2 activator (long-term safety)	Adolescents & Adults	Terminated (Phase 3)	Biogen
**HERA**	Lademirsen (anti-miR-21)	microRNA inhibitor	Adolescents & Adults	Terminated for futility	Sanofi
**DOUBLE PRO-TECT Alport**	Dapagliflozin	SGLT2 inhibitor	Adolescents & Young Adults	Recruiting (Phase 3)	German Research Foundation DFG

The original article has been corrected.

